# Gut-brain immune dysregulation in Parkinson’s disease: a hypothesis-forming narrative review of trained immunity

**DOI:** 10.3389/fnagi.2026.1973833

**Published:** 2026-09-23

**Authors:** Mingke Tang, Jingxian Li, Chuanzhang Zhang, Ruoxin Tu, Shun Wang

**Affiliations:** 1 Heilongjiang Academy of Traditional Chinese Medicine, Harbin, China; 2 Heilongjiang University of Chinese Medicine, Harbin, China; 3 Heilongjiang University of Chinese Medicine Affiliated Second Hospital, Harbin, China

**Keywords:** alpha-synuclein, gut microbiota, gut-brain axis, inflammaging, microglia, neuroinflammation, Parkinson’s disease, trained immunity

## Abstract

Parkinson’s disease (PD) is increasingly understood as a multisystem disorder in which gastrointestinal dysfunction, immune remodeling, protein aggregation, and central neurodegeneration interact across the disease course. Although gut microbiota alterations are repeatedly reported in PD, taxonomic differences alone do not explain how peripheral ecological signals acquire neurological relevance. This review therefore focuses on immune translation: the processes through which dysbiosis, microbial metabolites, intestinal barrier stress, and mucosal inflammation may reshape peripheral immunity and lower the threshold for central neuroinflammatory responses. Particular attention is given to trained immunity as a mechanistic framework that may connect repeated low-grade gut-derived stimulation with persistent innate immune reprogramming. Direct evidence that trained immunity drives PD remains limited; the concept is presented as a testable bridge rather than an established causal pathway. We integrate this framework with age-related immunosenescence and inflammaging, enteric nervous system vulnerability, vagal communication, alpha-synuclein pathology, blood–brain barrier signaling, and microglial priming. Human microbiome studies, Parkinsonian animal models, LPS-based innate immune-memory paradigms, and emerging intervention trials are considered according to their evidentiary level and major confounders, including constipation, diet, medication exposure, geography, and disease stage. The resulting model accommodates body-first, brain-first, and mixed trajectories rather than assigning a universal gut origin to PD. Therapeutically, the evidence supports a shift from nonspecific microbiome normalization toward mechanism-matched strategies that combine ecological, immune, metabolic, and clinical biomarkers. Longitudinal prodromal cohorts and integrated multi-omics with immune phenotyping will be essential to determine whether gut-brain immune signatures identify causal pathways, progression markers, or treatment-responsive subgroups.

## 1 Introduction

Parkinson’s disease (PD) is defined clinically by a progressive movement disorder and pathologically by degeneration within the nigrostriatal system together with alpha-synuclein-rich Lewy pathology. That brain-centered description remains indispensable, but it does not account well for the breadth or timing of non-motor manifestations. Constipation, autonomic dysfunction, sleep disturbance, altered gastrointestinal physiology, and systemic inflammatory changes can precede or accompany the motor syndrome. These observations have encouraged a multisystem view in which the gastrointestinal tract, immune system, enteric nervous system (ENS), autonomic pathways, vasculature, and central nervous system (CNS) interact over time (Chen et al., 2025; Wang et al., 2021; Zhao et al., 2026; Caradonna et al., 2024).

Age is the dominant background risk for sporadic PD and is also a biological modifier of nearly every component of the gut-brain axis. Aging alters intestinal epithelial resilience, microbial ecology, immune-cell composition, vascular function, proteostasis, and glial responsiveness. Immunosenescence and inflammaging can therefore change the consequences of a microbial or inflammatory exposure even when the exposure itself is not disease specific (Bertoni et al., 2026; Nguyen et al., 2023). For a journal focused on aging neuroscience, the relevant question is not simply whether the gut differs in PD, but how age-dependent loss of homeostatic reserve may convert peripheral perturbations into persistent neuroimmune stress.

The microbiome literature has produced an important but incomplete signal. Multiple cohorts report differences in gut bacterial composition in PD, yet the taxa that increase or decrease are not fully reproducible across populations. Early case–control studies and longitudinal work also show that constipation, medication exposure, geography, sequencing methods, and disease duration materially influence the observed profiles (Scheperjans et al., 2015; Keshavarzian et al., 2015; Hill-Burns et al., 2017; Aho et al., 2019). Thus, dysbiosis should not be treated as a single microbial signature. A more defensible interpretation is that different ecological configurations may converge on altered microbial functions, including short-chain fatty acid (SCFA) production, mucin metabolism, endotoxin exposure, bile acid transformation, and tryptophan metabolism (Xu et al., 2026; Czaj et al., 2026; Duan et al., 2024).

This functional view raises the central problem addressed here: how are ecological disturbances translated into host signals that can influence neurodegeneration? Intestinal barrier stress, pattern-recognition receptor signaling, mucosal immune activation, peripheral cytokines, T-cell responses, neurovascular signaling, and microglial priming offer plausible routes (Yuan et al., 2023; Gorecki et al., 2021; Campos-Acuna et al., 2019; Carloni and Rescigno, 2023; Cossu et al., 2023). We use the term immune translation for this intermediate layer. It is deliberately broader than systemic inflammation; it includes changes in immune set points, barrier interfaces, cell-state memory, and the context in which later stimuli are interpreted.

Within that framework, trained immunity is a particularly useful but still provisional concept. Trained immunity refers to persistent functional reprogramming of innate immune cells after an initial stimulus, mediated by coordinated epigenetic and metabolic changes rather than antigen-specific receptors (Netea et al., 2020; Saeed et al., 2014). Experimental work also demonstrates that long-lasting innate immune memory can occur in the brain and modify responses to subsequent neurological insults (Wendeln et al., 2018). These findings do not prove a gut-derived trained-immunity pathway in PD. They do, however, provide a mechanistic vocabulary for testing whether repeated low-grade microbial or inflammatory exposure leaves a durable peripheral or central immune imprint that interacts with aging and alpha-synuclein pathology.

As a hypothesis-forming narrative review, the aim is to synthesize PD gut-brain evidence around a sequence of ecological disturbance, immune translation, neural and vascular communication, and central amplification. We distinguish associative human findings from experimental causality and established clinical effects from therapeutic plausibility. This distinction is important because recent reviews already cover microbiota composition, gut-derived metabolites, alpha-synuclein propagation, and microbiome-directed therapy in considerable detail (Chen et al., 2025; Wang et al., 2021; Zhao et al., 2026; Auclair-Ouellet et al., 2025). The added value of the present framework is to place age-related immune remodeling and trained-immunity-like persistence at the interface between gut ecology and neuroinflammation, while retaining body-first, brain-first, and mixed disease trajectories as competing or complementary models rather than mutually exclusive claims.

## 2 Gut ecological dysregulation in Parkinson’s disease

### 2.1 Compositional heterogeneity and functional convergence

The strongest reproducible conclusion from PD microbiome studies is not the presence of one diagnostic taxon but the existence of ecological disturbance. Scheperjans and colleagues reported microbiota differences related to PD phenotype, and subsequent cohorts identified additional associations with bacterial groups involved in mucin degradation, SCFA production, and inflammatory regulation (Scheperjans et al., 2015; Keshavarzian et al., 2015). Larger analyses have repeatedly shown that medication use can leave a distinct microbial signature, complicating efforts to attribute changes exclusively to disease biology (Hill-Burns et al., 2017). Longitudinal observations further indicate that some microbial features persist whereas others vary with time and disease progression (Aho et al., 2019).

This heterogeneity should change how microbiome findings are interpreted. Relative abundance is affected by transit time, stool consistency, diet, geography, host genetics, age, sequencing platform, extraction methods, and statistical normalization. Constipation is especially important in PD because prolonged transit can alter substrate availability, luminal pH, oxygen gradients, and metabolite concentrations. It can therefore act simultaneously as a prodromal feature, a consequence of autonomic dysfunction, and a confounder of microbiome analyses. A taxon that appears disease associated may partly reflect gastrointestinal physiology rather than a primary pathogenic process (Wang et al., 2021; Zhao et al., 2026).

Functional convergence is a more robust organizing principle. Experimental data provide proof of principle that microbial communities can modify neurological phenotypes: in an alpha-synuclein-overexpressing mouse model, microbiota depletion and microbial reconstitution altered motor deficits and neuroinflammatory responses (Sampson et al., 2016). Such studies establish biological capacity rather than human causality. In patients, the relevant inference is more modest: PD-associated ecological shifts may change the production or exposure of molecules that regulate epithelial integrity, mucosal immunity, neural signaling, and glial thresholds.

### 2.2 Oral-gut interfaces and cumulative mucosal exposure

The intestinal microbiome is only one component of the mucosal ecosystem. Oral health, periodontal inflammation, salivary flow, swallowing, diet, medication-related xerostomia, and age-dependent changes in dental care can alter oral microbial communities. Repeated swallowing also creates continuous exposure of the upper gastrointestinal tract to oral organisms and microbial products. Reviews linking periodontal and intestinal inflammagens with PD emphasize the possibility that mucosal inflammatory burden is distributed across connected sites rather than confined to stool-derived signals (van Vuuren et al., 2021).

This broader view matters for immune translation. A patient with constipation, periodontal inflammation, epithelial barrier stress, and altered microbial metabolites may experience a different cumulative inflammatory input from a patient with the same motor severity but preserved gastrointestinal function. Such differences are unlikely to be captured by a single fecal sequencing sample. Multi-site sampling, including oral and stool specimens, could therefore be valuable in mechanistic phenotyping, particularly when paired with inflammatory markers and gastrointestinal measures. The evidence is not yet sufficient to define an oral-gut PD subtype, but the concept highlights a recurring limitation of current studies: they often isolate one mucosal compartment from a system that is biologically continuous.

### 2.3 Microbial metabolites as functional mediators

Microbial metabolites offer a more direct bridge between community composition and host physiology. SCFAs, tryptophan-derived metabolites, bile acid derivatives, and microbial-associated molecular patterns can affect epithelial cells, immune populations, enteric neurons, vascular interfaces, and microglia (Czaj et al., 2026; Mu and Wang, 2025; Eicher and Mohajeri, 2022). Their effects are context dependent, and the same metabolite can have different consequences according to concentration, tissue compartment, receptor expression, and inflammatory state.

SCFAs illustrate this complexity. Butyrate is a major energy source for colonocytes and can support epithelial barrier integrity, regulatory immune responses, and histone acetylation. Clinical studies have reported differences in fecal SCFAs and SCFA-associated microbial groups in PD (Unger et al., 2016). Yet fecal concentration is not equivalent to mucosal production, portal exposure, or systemic bioavailability. Reduced fecal butyrate cannot therefore be interpreted automatically as a causal deficiency. Conversely, experimental work has shown that microbial metabolites can modulate neuroinflammation in alpha-synuclein models, underscoring that SCFA biology cannot be reduced to a uniformly protective or harmful label (Sampson et al., 2016). The more useful question is which SCFA profile, in which host context, modifies barrier and immune function.

Tryptophan metabolism provides a second mechanistic axis. Microbial and host pathways generate indoles, kynurenine metabolites, serotonin-related products, and aryl hydrocarbon receptor ligands. These compounds can influence epithelial tolerance, oxidative stress, immune differentiation, and neural function (Czaj et al., 2026; Mu and Wang, 2025). Bile acid metabolism similarly links microbial transformation with metabolic and inflammatory signaling. Both pathways remain less clinically standardized than stool taxonomic profiling, but they may ultimately prove more interpretable because they measure biochemical function rather than community membership alone.

Microbial products such as lipopolysaccharide (LPS) are particularly relevant to chronic innate immune stimulation. Barrier-permeating exposure to microbial-associated molecular patterns need not produce overt infection to alter host signaling. Repeated, low-amplitude stimulation of pattern-recognition receptors could change monocyte, macrophage, or tissue-resident immune responses over time (Yuan et al., 2023; Gorecki et al., 2021). This possibility provides a conceptual transition from dysbiosis to trained immunity, but direct demonstration of durable gut-derived innate immune training in PD patients remains an important evidence gap.

### 2.4 Intestinal barrier dysfunction and local immune activation

The intestinal barrier is the anatomical interface at which ecological change becomes host exposure. Its function depends on epithelial tight junctions, mucus, antimicrobial peptides, secretory immunoglobulin A, stromal support, immune tolerance, and microbial metabolism. Barrier dysfunction has been discussed in PD in association with gastrointestinal inflammation, constipation, altered microbial profiles, and enteric alpha-synuclein pathology (Wang et al., 2021; Zhao et al., 2026; Gorecki et al., 2021). Importantly, barrier integrity is dynamic. Aging, diet, infection, medication, metabolic disease, and transit abnormalities can modify permeability independently of PD.

When barrier function is compromised, microbial products gain greater access to epithelial and immune compartments. Macrophages, dendritic cells, mast cells, innate lymphoid cells, and epithelial signaling pathways can then generate cytokines, chemokines, and reactive mediators. Mast-cell activation is one potential contributor to gut-brain inflammatory signaling, although evidence specific to PD remains less developed than for macrophage- and T-cell-mediated pathways (Boziki et al., 2024). These local signals can alter enteric neuronal function and may also enter the systemic circulation or influence neural afferents.

Aging may amplify this transition by reducing epithelial repair capacity and altering immune tolerance. Rather than assuming a binary ‘leaky gut’ phenotype, future studies should measure barrier function alongside stool consistency, transit time, inflammatory mediators, microbial products, and age-related comorbidity. Such integrated assessment would help determine whether permeability is a cause, a modifier, or a consequence of PD-related gastrointestinal dysfunction.

### 2.5 Biomarkers and patient stratification

The diagnostic performance of individual microbiota markers remains limited by cohort specificity. A more realistic clinical role may be stratification rather than diagnosis. Composite profiles could combine microbial functions, metabolites, constipation severity, autonomic symptoms, inflammatory markers, medication exposure, diet, and alpha-synuclein-related measurements (Khan et al., 2026; Liu et al., 2022). This approach is consistent with the biological heterogeneity of PD and avoids treating a microbial signature as a stand-alone disease test.

The intended use of a biomarker should also be explicit. A feature associated with constipation-dominant PD may be useful for selecting a bowel-directed intervention but not for predicting conversion from prodromal disease. Conversely, an inflammatory-metabolic profile that precedes motor diagnosis would have different implications for risk stratification. Longitudinal studies are therefore essential. Repeated sampling can distinguish stable traits from state-dependent changes related to medication, diet, intercurrent illness, or disease progression (Aho et al., 2019).

## 3 Immune translation: from gut-derived signals to trained immunity

### 3.1 Pattern-recognition signaling and mucosal immune activation

Immune translation begins when ecological signals are sensed by host cells. Toll-like receptors (TLRs), inflammasome-associated pathways, cytokine receptors, and other pattern-recognition systems integrate microbial products with endogenous danger signals. TLR2 and TLR4 are of particular interest in PD because they can respond to microbial ligands and to damage-associated cues linked to alpha-synuclein and cellular stress (Gorecki et al., 2021). Their downstream effects include NF-kappaB-dependent transcription, cytokine release, and interactions with inflammasome signaling. These pathways should not be presented as a single linear cascade; they form a context-dependent network shaped by ligand dose, prior exposure, cell type, and aging.

At the mucosal level, chronic low-grade stimulation may be more relevant than acute systemic inflammation. Persistent exposure can alter macrophage and dendritic-cell phenotypes, regulatory networks, and the balance between tolerance and reactivity. Gut-derived cytokines and microbial products can also affect endothelial cells and peripheral leukocytes, creating routes by which a localized intestinal disturbance influences systemic immune tone (Yuan et al., 2023; Carloni and Rescigno, 2023; Zhao et al., 2025). This provides a biologically plausible bridge to the CNS without requiring large amounts of microbial material to enter the brain.

### 3.2 Trained immunity as a persistence mechanism

Trained immunity describes a form of innate immune memory in which an initial stimulus produces lasting changes in subsequent responsiveness. Unlike adaptive immune memory, it does not depend on antigen-specific lymphocyte receptors. Instead, metabolic and epigenetic remodeling can alter enhancer accessibility, transcriptional programs, and cellular metabolism in monocytes, macrophages, and progenitor populations (Netea et al., 2020; Saeed et al., 2014). The concept is particularly relevant to chronic diseases in which repeated inflammatory exposures occur over years rather than as a single discrete event.

For PD, trained immunity is best treated as a hypothesis-generating framework rather than an established mechanism. Gut dysbiosis, microbial products, altered SCFAs, periodontal inflammation, infection history, metabolic disease, and aging could provide repeated innate stimuli. If those exposures produced durable reprogramming in circulating monocytes or tissue macrophages, later encounters with alpha-synuclein species or neuronal injury might elicit exaggerated responses. Aging could further bias this system through inflammaging and reduced capacity to return to immune homeostasis (Bertoni et al., 2026; Cossu et al., 2023; Freuchet et al., 2024).

The framework also offers an explanation for persistence. A transient episode of barrier dysfunction would not need to remain continuously active if it left a longer-lived immune imprint. It could also help explain convergence: different upstream exposures might generate partially overlapping inflammatory phenotypes. However, evidence for these possibilities in PD is currently indirect. Studies are needed to test whether patients with defined gut abnormalities exhibit stable epigenetic or metabolic signatures of innate immune training in monocytes, whether those signatures persist after adjustment for age and medication, and whether they predict neuroinflammatory biomarkers or disease progression.

Innate immune memory is also relevant within the CNS. Experimental work has shown that microglial responses can be durably altered by prior systemic inflammatory exposures, with consequences for later neurological pathology (Wendeln et al., 2018). This does not mean that peripheral trained immunity and microglial memory are the same process. Rather, they may represent interacting levels of immune history. A rigorous PD model should therefore distinguish peripheral immune training, endothelial or vascular priming, and microglial state changes rather than grouping them under a single label.

### 3.3 Peripheral cytokines, T cells, and systemic immune tone

Adaptive immunity adds another layer to the gut-brain immune axis. T-cell abnormalities and alpha-synuclein-reactive T cells have been reported in PD, supporting the possibility that protein pathology can become immunologically visible (Campos-Acuna et al., 2019; Sulzer et al., 2017). These findings do not establish that gut-derived antigens initiate the response. They do show that peripheral immune recognition of disease-relevant proteins is biologically plausible and may interact with innate pathways.

Systemic cytokines can influence the CNS through several mechanisms: endothelial signaling, changes in blood–brain barrier transport or permeability, activation of perivascular macrophages, communication through circumventricular regions, and neural or neuroendocrine pathways (Carloni and Rescigno, 2023; Cossu et al., 2023). In an aging host, vascular and immune reserve may already be reduced, potentially increasing the impact of modest peripheral inflammatory changes. The important mechanistic issue is therefore not whether a cytokine physically crosses the blood–brain barrier in large quantities, but whether the neurovascular unit receives and amplifies peripheral inflammatory information (see Table 1).

**Table 1:** Mechanistic evidence map for the gut-brain immune axis in Parkinson’s disease.

Mechanistic layer	Representative evidence	Aging relevance	Main uncertainty
Gut ecological disturbance	Human cohorts repeatedly identify compositional and functional differences, but taxonomic signatures vary across populations.	Aging, diet, constipation, and polypharmacy reshape the microbiome.	Driver versus consequence; limited cross-cohort reproducibility.
Microbial metabolites	SCFAs, tryptophan metabolites, bile acids, and microbial products can modify epithelial and immune signaling.	Age-related metabolic and epithelial changes may alter tissue exposure and response.	Stool concentration does not equal mucosal or systemic exposure.
Barrier and mucosal immunity	Barrier stress can increase contact between microbial products and innate immune cells.	Epithelial repair and immune tolerance decline with age.	Few studies measure permeability, microbiota, and immune states together.
Peripheral immune reprogramming	TLR signaling, cytokines, and T-cell responses provide plausible systemic routes.	Inflammaging may raise the basal inflammatory set point.	Causality and temporal ordering are uncertain.
Trained immunity	Epigenetic and metabolic innate memory is well established outside PD and offers a persistence mechanism.	Aging may interact with accumulated inflammatory exposures.	Direct gut-to-trained-immunity evidence in PD is sparse.
ENS/vagal signaling and alpha-synuclein	Rodent studies demonstrate gut-to-brain propagation under experimental conditions; human universality is unresolved.	ENS vulnerability and autonomic dysfunction increase with age.	Body-first, brain-first, and mixed trajectories likely coexist.
Neurovascular signaling and microglia	Peripheral signals can act through endothelium, perivascular cells, and primed microglia.	Vascular aging and microglial priming may amplify responses.	Need longitudinal target-engagement biomarkers.

## 4 Enteric nervous system, vagal signaling, and alpha-synuclein pathology

### 4.1 Enteric neuronal vulnerability in an aging mucosal environment

The ENS is positioned at the intersection of microbial, immune, and neural biology. Enteric neurons and glia are embedded within a tissue exposed to epithelial signals, immune mediators, mechanical forces, and microbial metabolites. Aging changes ENS structure and function and may reduce resilience to oxidative, inflammatory, and metabolic stress (Nguyen et al., 2023; Valdetaro et al., 2026). In PD, this interface is relevant because constipation and autonomic dysfunction can precede motor diagnosis and because alpha-synuclein pathology has been described in peripheral neural tissues.

Inflammatory stress could modify alpha-synuclein expression, post-translational modification, aggregation, or clearance in enteric neurons. Microbial metabolites can also alter mitochondrial function and neuronal excitability. These observations make the gut a plausible site where ecological and immune perturbations interact with proteostasis. They do not establish that enteric alpha-synuclein is the initiating lesion in most patients. Peripheral pathology may be primary in some individuals, parallel to central pathology in others, or secondary to established disease.

### 4.2 The vagus nerve as a communication route

The vagus nerve provides a major bidirectional connection between visceral organs and the brainstem. It transmits sensory information, participates in inflammatory reflexes, and links peripheral physiological states with central autonomic networks (Ma et al., 2025). Gut-brain communication through the vagus therefore extends beyond physical transport of protein aggregates. Cytokines, enteroendocrine signals, metabolites, and local immune-neural interactions can alter vagal activity without requiring alpha-synuclein to move between neurons.

Experimental studies nevertheless support the biological possibility of neural propagation. Misfolded alpha-synuclein introduced into the gastrointestinal tract can reach the brain in rodent models, and gut-seeded pathology has been shown to spread through anatomically connected regions; vagotomy can interrupt this process in specific experimental paradigms (Holmqvist et al., 2014; Kim et al., 2019). These studies are important mechanistic evidence, but they do not demonstrate that the same sequence is universal in sporadic human PD. Experimental inoculation also differs substantially from spontaneous disease in dose, molecular species, anatomical site, and timing.

### 4.3 Body-first and brain-first trajectories

The most useful clinical interpretation is therefore not that ‘PD begins in the gut’ but that gut-related mechanisms may contribute strongly in biologically defined subgroups. Body-first models are compatible with early autonomic, gastrointestinal, or sleep-related involvement, whereas brain-first models place initial pathology within the CNS. Mixed trajectories are also likely (Zhao et al., 2026; Caradonna et al., 2024; Freuchet et al., 2024). This heterogeneity helps reconcile apparently conflicting data on enteric alpha-synuclein, vagotomy, constipation, and central imaging.

An immune-centered framework adds another dimension to these trajectories. A patient could have a body-first neural pattern with little systemic inflammation, or a brain-first pattern accompanied by substantial gut dysbiosis that modifies progression rather than onset. Future studies should therefore avoid using gastrointestinal symptoms alone as a proxy for a gut-origin mechanism. Mechanistic subtyping will require combinations of autonomic measures, microbiota and metabolite data, immune phenotyping, alpha-synuclein biomarkers, and longitudinal imaging or clinical outcomes.

## 5 Microglial priming and neuroinflammatory amplification

### 5.1 Peripheral-to-central immune communication

Microglia are central immune sentinels that respond to protein aggregates, neuronal injury, cytokines, vascular signals, metabolites, and age-related tissue changes. In PD, they are well positioned to integrate peripheral inflammatory information with alpha-synuclein pathology and dopaminergic neuronal stress (Cossu et al., 2023; Arena et al., 2022). Gut-derived signals do not need to directly injure substantia nigra neurons to be relevant. A more plausible mechanism is that peripheral inflammation changes the threshold, duration, or resolution of central immune responses.

The blood–brain barrier and neurovascular unit are critical intermediates. Endothelial cells, pericytes, astrocytic end-feet, and perivascular macrophages regulate immune signaling at the CNS interface. Peripheral cytokines and microbial products may modify endothelial activation or transport even when barrier integrity is not globally disrupted (Carloni and Rescigno, 2023). Age-related vascular dysfunction can further alter this response. This vascular perspective avoids an overly simplistic model in which gut-derived molecules must freely cross a ‘leaky’ blood–brain barrier to affect the brain.

### 5.2 Alpha-synuclein and inflammation as a feed-forward loop

Alpha-synuclein and inflammation can reinforce one another. Aggregated or modified alpha-synuclein can activate innate immune pathways, whereas inflammatory and oxidative stress can impair proteostasis, mitochondrial function, and clearance mechanisms (Cossu et al., 2023; Arena et al., 2022). Microglia are central to this loop because they respond to extracellular alpha-synuclein species and neuronal damage while also shaping the local cytokine and redox environment.

Peripheral priming could intensify this feedback. If aging, infection, metabolic stress, or chronic mucosal inflammation has already shifted innate immune set points, subsequent alpha-synuclein-related injury may produce a larger or less resolving response. The trained-immunity framework provides one possible explanation for such history dependence, while experimental microglial-memory studies show that prior inflammatory events can alter later CNS responses (Netea et al., 2020; Wendeln et al., 2018). Direct validation in PD, however, requires longitudinal measurement of immune states before and after clinically meaningful transitions.

### 5.3 Neuroinflammatory biomarkers and stratified risk

A major translational challenge is identifying patients in whom neuroinflammation is biologically important rather than merely detectable. Candidate markers include peripheral cytokines, immune-cell phenotypes, plasma or cerebrospinal fluid proteins, neuroimaging signals, microbial metabolites, and gut ecological features (Liu et al., 2022). No single marker captures the entire process, and many inflammatory measures are influenced by age, adiposity, infection, medication, sleep, and comorbidity.

Multi-domain panels may therefore be more useful than single analytes. Conceptually, patients could be described by combinations such as dysbiosis-high, barrier-stress-high, peripheral-inflammation-high, or central-neuroinflammation-high. These labels are not validated clinical subtypes, but they illustrate how mechanism-linked stratification could improve trial design. A gut-directed intervention may fail in an unselected PD population even if it is effective in a subgroup with a demonstrable gut-immune disturbance.

## 6 Therapeutic opportunities along the gut-brain immune axis

### 6.1 Diet, prebiotics, probiotics, and synbiotics

Diet is a major determinant of microbial metabolism and intestinal physiology. Fiber, polyphenols, fat quality, protein source, and fermented foods can alter substrate availability and microbial metabolite production. In PD, dietary intervention also intersects with constipation, frailty, weight loss, and levodopa-protein timing. The therapeutic objective should therefore be functional: improving bowel function, barrier resilience, or metabolite profiles rather than attempting to normalize the microbiome toward a universal reference state (2, 22).

Probiotics and prebiotics are attractive because of their relative accessibility, but their mechanisms are strain- and substrate-specific. A randomized placebo-controlled trial showed that multistrain probiotics can improve constipation-related outcomes in PD (Tan et al., 2021). This is clinically relevant but should not be equated with disease modification or reduced neuroinflammation. Trials that aim to test a neuroimmune mechanism need biomarker endpoints in addition to gastrointestinal symptoms.

Synbiotic strategies may offer complementary effects by combining microbial strains with fermentable substrates. Yet baseline microbiota, diet, bowel transit, and medication use are likely to influence response. Future trials should predefine the biological target of the intervention and measure whether that target changes. Without such mechanistic anchoring, a positive gastrointestinal outcome cannot establish that the gut-brain immune axis has been modified.

### 6.2 Fecal microbiota transplantation

Fecal microbiota transplantation (FMT) attempts to modify the intestinal ecosystem at community level rather than through selected strains. Its rationale in PD includes effects on diversity, metabolite production, bowel function, barrier signaling, and inflammatory tone. Controlled clinical trials have now moved the field beyond isolated case reports, but the results are not uniform. The GUT-PARFECT phase 2 trial reported a signal of clinical benefit after a single FMT in mild-to-moderate PD, while other randomized work has underscored uncertainty about the magnitude and reproducibility of motor effects (Bruggeman et al., 2024; Scheperjans et al., 2024). These data support continued investigation rather than routine clinical use.

FMT also raises practical and biological questions: donor selection, route of administration, durability of engraftment, antimicrobial resistance, pathogen screening, recipient diet, and the possibility that a transplanted community has different effects in different host immune environments. Mechanistic trials should therefore pair clinical outcomes with metagenomics, metabolomics, barrier markers, and immune phenotyping. A durable clinical effect without evidence of ecological or immune change would require a different interpretation from a biomarker-linked response.

### 6.3 Metabolite- and barrier-targeted approaches

Targeting microbial function may ultimately be more precise than altering community composition. SCFA-related strategies, tryptophan-pathway modulation, bile acid signaling, and interventions that support epithelial integrity are all plausible options (Xu et al., 2026; Duan et al., 2024; Mu and Wang, 2025). Their appeal lies in defined biochemical targets, but the same caution applies: circulating or stool metabolite concentrations may not reflect tissue exposure, and effects can be receptor- and context-dependent.

Barrier support is best viewed as a therapeutic objective rather than a single treatment class. Dietary fiber, treatment of constipation, management of gastrointestinal comorbidity, and reduction of avoidable mucosal stress may all contribute. The key trial-design question is whether an intervention demonstrably improves barrier function and whether that change is accompanied by altered immune signaling. Without such paired measures, claims of gut-brain mechanism remain speculative.

### 6.4 Immune modulation and combination strategies

Because immune dysregulation lies at the center of the proposed framework, direct immune modulation is an obvious but more hazardous therapeutic concept. Potential targets include excessive TLR signaling, inflammasome activity, regulatory immune imbalance, maladaptive innate immune memory, or microglial overactivation (Gorecki et al., 2021; Campos-Acuna et al., 2019; Cossu et al., 2023). Broad immunosuppression is unlikely to be appropriate for chronic PD because host defense and tissue repair must be preserved. Any immune-directed strategy will require better biological selection than is currently available.

Combination approaches may be more realistic. A patient with marked constipation, dysbiosis, reduced SCFA-related function, and peripheral inflammation may require bowel management, dietary optimization, targeted microbiota modulation, and monitoring of immune markers rather than a single intervention. In contrast, a patient without measurable gut-immune abnormalities may derive little benefit from microbiome-directed treatment. The translational goal is therefore mechanism matching, not universal gut therapy (see Table 2).

**Table 2:** Therapeutic strategies mapped to proposed gut-brain immune targets.

Strategy	Primary target	Current evidence maturity	Key limitations
Dietary optimization	Microbial substrates, bowel function, metabolite production, barrier resilience	Clinically feasible; PD-specific disease-modifying evidence remains limited.	Dietary adherence, frailty, levodopa-protein timing, heterogeneous baseline microbiota.
Prebiotics/probiotics/synbiotics	Microbial function, SCFAs, constipation, epithelial signaling	Randomized evidence supports improvement of constipation with multistrain probiotics.	Gastrointestinal benefit does not establish neuroimmune or disease-modifying effect.
FMT	Community-level ecological remodeling and metabolite output	Randomized trials show feasibility and an emerging but inconsistent clinical signal.	Donor selection, route, durability, safety, host dependence, uncertain mechanism.
Metabolite/barrier approaches	SCFAs, tryptophan/bile-acid pathways, epithelial integrity	Mechanistically attractive; human PD evidence is early.	Context-dependent metabolite effects and uncertain tissue exposure.
Immune modulation	TLR/inflammasome signaling, regulatory balance, maladaptive innate memory, microglial activation	Predominantly mechanistic/preclinical for gut-brain applications.	Safety, target specificity, need for biomarker-defined patient selection.
Combination precision strategies	Multiple nodes selected according to patient phenotype and biomarkers	Conceptual/early translational stage.	Requires validated subtypes and target-engagement markers.

## 7 Discussion

### 7.1 An integrated age-sensitive gut-brain immune model

The evidence considered here supports a layered model rather than a single causal chain. Gut and oral ecological disturbances may alter microbial metabolites and inflammatory exposure. Barrier stress and mucosal immunity can translate those changes into systemic signals. Peripheral immune states interact with the ENS, vagal pathways, and the neurovascular unit, while alpha-synuclein pathology and microglial activation create central amplification. Aging modifies each layer through immunosenescence, inflammaging, barrier decline, vascular vulnerability, and reduced proteostatic reserve (Bertoni et al., 2026; Nguyen et al., 2023; Carloni and Rescigno, 2023). The result is a network in which similar clinical PD phenotypes may arise from different combinations of peripheral and central mechanisms.

This model differs from a simple microbiome hypothesis in two ways. First, it treats the microbiota as one source of signals rather than the sole driver of disease. Second, it introduces immune history as a potential determinant of response. Trained immunity provides a plausible explanation for why repeated peripheral stimulation might have consequences that outlast the original exposure (Netea et al., 2020; Saeed et al., 2014). Microglial innate memory provides a parallel CNS concept (Wendeln et al., 2018). Neither mechanism has yet been demonstrated as a necessary step in human PD, and that uncertainty should remain explicit.

### 7.2 Causality and disease heterogeneity

Causality is the principal unresolved issue. Most human microbiome studies are cross-sectional, and even longitudinal associations can be difficult to interpret because constipation, medication, diet, and disease progression change simultaneously. Experimental models show that microbiota can modify neuroinflammation and motor phenotypes (Sampson et al., 2016), but model-specific manipulations do not establish a universal human pathway. The strongest future evidence would come from prodromal cohorts in which ecological, metabolic, immune, and neurological variables are measured before diagnosis and followed through conversion or non-conversion.

Disease origin is similarly heterogeneous. Gut-to-brain alpha-synuclein propagation is supported by compelling experimental work (Holmqvist et al., 2014; Kim et al., 2019), yet the human data do not justify a universal gut-first model. Body-first and brain-first trajectories can coexist, and the role of the gut may differ between disease initiation and progression. A gut-derived inflammatory signal could accelerate central pathology without being the site of first alpha-synuclein aggregation. This distinction is essential when interpreting both mechanistic studies and therapeutic trials.

### 7.3 Experimental support from parkinsonian animal models: microbiota, LPS, and microglial immune memory

Animal models provide an important intermediate level of evidence because they permit temporal and mechanistic manipulation that is not feasible in patients. In alpha-synuclein-overexpressing mice, microbiota depletion and reconstitution can alter motor abnormalities and microglial activation, establishing that microbial communities are capable of modifying Parkinsonian phenotypes under defined experimental conditions (Sampson et al., 2016). Across a broader preclinical literature, a systematic review of 29 murine studies found that gut microbiota-targeted interventions—including antibiotics, probiotics, prebiotics, synbiotics, and fecal microbiota transfer—most often reduced dopaminergic cell loss and glial activation while improving motor performance (Panaitescu et al., 2024). These findings strengthen biological plausibility, but they should not be read as evidence that a single dysbiotic configuration causes human PD: toxin-based, transgenic, and alpha-synuclein-seeding models reproduce different components of the disease and frequently use short intervention windows.

LPS paradigms are particularly informative for the trained-immunity hypothesis because the direction of subsequent responses depends on dose, timing, repetition, and cellular compartment. Low-dose peripheral LPS priming can establish a trained microglial state without requiring overt blood–brain barrier disruption. In a PD-relevant model, peripheral myeloid GSDMD signaling promoted microglial immune training through an IL-1R-dependent pathway and aggravated neuroinflammation and neurodegeneration after a subsequent Parkinsonian challenge (Wang et al., 2023). Conversely, repeated LPS exposure can also produce immune tolerance rather than amplification. Dong et al. showed that distinct LPS paradigms generated training or tolerance in the nigrostriatal system; microglial tolerance persisted for weeks and protected dopaminergic neurons in MPTP-treated mice, whereas HIF-1alpha-dependent innate immune memory influenced the magnitude of neuroinflammation (Dong et al., 2024). Thus, “LPS exposure” should not be treated as a uniform inflammatory variable.

These experiments provide contextual support for our proposed gut-to-immune translation framework in two ways. First, they show that peripheral inflammatory history can durably alter microglial responsiveness, making immune-state history a measurable biological variable rather than a purely theoretical construct. Second, they demonstrate that the same innate system can be trained toward heightened responsiveness or tolerized toward a dampened response depending on the exposure paradigm. This dose- and history-dependence is relevant to chronic gut-derived stimulation, which is more likely to consist of repeated low-amplitude microbial and barrier signals than of a single high-dose systemic endotoxin exposure. A critical next step is therefore to connect gut-origin exposures to these immune-memory phenotypes directly—for example, by combining controlled microbiota manipulation with monocyte epigenetic profiling, microglial-state analysis, alpha-synuclein pathology, and longitudinal behavioral outcomes in aged PD models.

### 7.4 Evidence limitations

Several limitations constrain the current field. Microbiome studies vary in sampling, sequencing, geography, diet assessment, medication adjustment, and constipation phenotyping. Metabolomic measurements differ by matrix and analytic platform. Peripheral inflammatory markers are often nonspecific and may reflect comorbidity rather than PD. Mechanistic links are usually studied in separate cohorts, so dysbiosis, barrier dysfunction, immune reprogramming, alpha-synuclein biology, and neuroinflammation are rarely demonstrated in the same individuals.

The evidence for trained immunity in PD is particularly preliminary. Core trained-immunity mechanisms are well established in other immunological contexts (Netea et al., 2020; Saeed et al., 2014), and innate immune memory can alter CNS responses experimentally (Wendeln et al., 2018), but direct PD studies linking gut exposures to durable monocyte or microglial epigenetic reprogramming remain sparse. Accordingly, trained immunity should currently be used to generate falsifiable hypotheses, not as a label for any chronic inflammation observed in PD.

This narrative review also has methodological limitations. It prioritizes mechanistic coherence and recent evidence rather than claiming a systematic search or quantitative synthesis. The literature is rapidly expanding, and several topics—including virome and mycobiome contributions, sex-specific effects, host genetics, and medication-microbiome pharmacology—are only briefly addressed. Conclusions should therefore be interpreted as a framework for targeted investigation rather than a comprehensive catalog of all gut-brain findings.

### 7.5 Priorities for future research

Longitudinal prodromal cohorts should become the central design for testing causality. Repeated stool and oral sampling should be paired with transit time, diet, medication, metabolomics, barrier markers, immune-cell phenotyping, autonomic measures, and validated alpha-synuclein or neurodegeneration biomarkers. Such datasets would make it possible to distinguish stable susceptibility traits from disease-state markers and treatment effects (Aho et al., 2019; Khan et al., 2026).

The trained-immunity hypothesis requires specific assays. Candidate studies should test chromatin accessibility, histone modifications, transcriptional programs, and immunometabolic states in circulating monocytes and, where feasible, tissue macrophages. These measures should be related prospectively to microbial products, SCFAs, barrier function, age, and inflammatory events. Demonstrating that a putative trained state predicts a standardized secondary response would be more informative than measuring resting cytokine concentrations alone.

Clinical trials should also move toward mechanism-enriched enrollment. Microbiota-targeted interventions could preselect patients by constipation phenotype, microbial function, metabolite profile, or barrier markers; immune-directed trials could use inflammatory or cellular signatures. Outcomes should include target engagement and not only motor scales. This approach may reduce dilution of biological effects in heterogeneous populations and clarify whether a therapy changes symptoms, disease biology, or both.

Finally, gut-brain immune models must be integrated with established PD biology, including genetics, mitochondrial dysfunction, lysosomal impairment, proteostasis, environmental exposures, and aging. The gut-brain axis is unlikely to replace these mechanisms. Its value is as an interacting layer that may alter susceptibility, timing, and progression in specific patients.

## 8 Conclusion

Gut-brain research in PD has progressed beyond the observation that patients and controls differ in stool microbiota. The more consequential question is how ecological disturbances are translated into host processes capable of modifying neural vulnerability. Current evidence supports biologically plausible links among microbial function, intestinal barrier stress, mucosal and systemic immunity, ENS and vagal signaling, alpha-synuclein pathology, neurovascular communication, and microglial activation. It does not establish a single gut-derived causal pathway for all PD.

Trained immunity offers a useful framework for the persistence and history dependence of these interactions, particularly in the context of aging and inflammaging. Its role in PD remains to be demonstrated directly in humans, although LPS-based and PD animal models now provide mechanistic support for immune-history-dependent microglial responses (Wang et al., 2023; Dong et al., 2024). The next phase of the field should therefore emphasize longitudinal human studies, integrated multi-omics, explicit immune-state measurements, and mechanism-matched trials. If those approaches identify reproducible gut-immune subgroups, the gut-brain axis may become clinically useful not as a universal explanation for PD, but as a basis for stratifying risk, progression, and treatment response.

## References

[ref1] Aho V. T. E. Pereira P. A. B. Voutilainen S. Paulin L. Pekkonen E. Auvinen P. . (2019). Gut microbiota in Parkinson's disease: temporal stability and relations to disease progression. EBioMedicine 44, 691–707. doi: 10.1016/j.ebiom.2019.05.064, 31221587 PMC6606744

[ref2] Arena G. Sharma K. Agyeah G. Kruger R. Grunewald A. Fitzgerald J. C. (2022). Neurodegeneration and neuroinflammation in Parkinson's disease: a self-sustained loop. Curr. Neurol. Neurosci. Rep. 22, 427–440. doi: 10.1007/s11910-022-01207-5, 35674870 PMC9174445

[ref3] Auclair-Ouellet N. Kassem O. Bronner S. Oula M. Binda S. (2025). Leveraging microbiome-based interventions to improve the management of neurodegenerative diseases: evidence for effects along the microbiota-gut-brain axis. Front. Nutr. 12:1699884. doi: 10.3389/fnut.2025.1699884, 41459056 PMC12739961

[ref4] Bertoni G. Ristori S. Monti D. (2026). Immunosenescence and inflammaging as drivers of neurodegeneration: cellular mechanisms, neuroimmune crosstalk, and therapeutic implications. Cells 15:657. doi: 10.3390/cells15080657, 42041525 PMC13114741

[ref5] Boziki M. Theotokis P. Kesidou E. Nella M. Bakirtzis C. Karafoulidou E. . (2024). Impact of mast cell activation on neurodegeneration: a potential role for gut-brain axis and *Helicobacter pylori* infection. Neurol. Int. 16, 1750–1778. doi: 10.3390/neurolint16060127, 39728753 PMC11677782

[ref6] Bruggeman A. Vandendriessche C. Hamerlinck H. de Looze D. Tate D. J. Vuylsteke M. . (2024). Safety and efficacy of faecal microbiota transplantation in patients with mild to moderate Parkinson's disease (GUT-PARFECT): a double-blind, placebo-controlled, randomised, phase 2 trial. EClinicalMedicine 71:102563. doi: 10.1016/j.eclinm.2024.102563, 38686220 PMC11056595

[ref7] Campos-Acuna J. Elgueta D. Pacheco R. (2019). T-cell-driven inflammation as a mediator of the gut-brain axis involved in Parkinson's disease. Front. Immunol. 10:239. doi: 10.3389/fimmu.2019.00239, 30828335 PMC6384270

[ref8] Caradonna E. Nemni R. Bifone A. Gandolfo P. Costantino L. Giordano L. . (2024). The brain-gut axis, an important player in Alzheimer and Parkinson disease: a narrative review. J. Clin. Med. 13:4130. doi: 10.3390/jcm13144130, 39064171 PMC11278248

[ref9] Carloni S. Rescigno M. (2023). The gut-brain vascular axis in neuroinflammation. Semin. Immunol. 69:101802. doi: 10.1016/j.smim.2023.101802, 37422929

[ref10] Chen C. Wang G. Q. Li D. D. Zhang F. (2025). Microbiota-gut-brain axis in neurodegenerative diseases: molecular mechanisms and therapeutic targets. Mol Biomed. 6:64. doi: 10.1186/s43556-025-00307-1, 40952592 PMC12436269

[ref11] Cossu D. Hatano T. Hattori N. (2023). The role of immune dysfunction in Parkinson's disease development. Int. J. Mol. Sci. 24:16766. doi: 10.3390/ijms242316766, 38069088 PMC10706591

[ref12] Czaj P. V. Szewczyk-Golec K. Nuszkiewicz J. Wozniak A. (2026). Gut dysbiosis and microbiota-derived metabolites in neurodegenerative diseases: molecular and biochemical mechanisms along the gut-brain axis. Molecules 31:490. doi: 10.3390/molecules31030490, 41683467 PMC12899277

[ref13] Dong H. Zhang X. Duan Y. He Y. Zhao J. Wang Z. . (2024). Hypoxia inducible factor-1alpha regulates microglial innate immune memory and the pathology of Parkinson's disease. J. Neuroinflammation 21:80. doi: 10.1186/s12974-024-03070-2 PMC10981320 38555419

[ref14] Duan W. X. Wang F. Liu J. Y. Liu C. F. (2024). Relationship between short-chain fatty acids and Parkinson's disease: a review from pathology to clinic. Neurosci. Bull. 40, 500–516. doi: 10.1007/s12264-023-01123-9, 37755674 PMC11003953

[ref15] Eicher T. P. Mohajeri M. H. (2022). Overlapping mechanisms of action of brain-active bacteria and bacterial metabolites in the pathogenesis of common brain diseases. Nutrients 14:2661. doi: 10.3390/nu14132661, 35807841 PMC9267981

[ref16] Freuchet A. Pincon A. Sette A. Lindestam Arlehamn C. S. (2024). Inflammation and heterogeneity in synucleinopathies. Front. Immunol. 15:1432342. doi: 10.3389/fimmu.2024.1432342, 39281666 PMC11392857

[ref17] Gorecki A. M. Anyaegbu C. C. Anderton R. S. (2021). TLR2 and TLR4 in Parkinson's disease pathogenesis: the environment takes a toll on the gut. Transl. Neurodegener. 10:47. doi: 10.1186/s40035-021-00271-0, 34814947 PMC8609261

[ref18] Hill-Burns E. M. Debelius J. W. Morton J. T. Wissemann W. T. Lewis M. R. Wallen Z. D. . (2017). Parkinson's disease and Parkinson's disease medications have distinct signatures of the gut microbiome. Mov. Disord. 32, 739–749. doi: 10.1002/mds.26942, 28195358 PMC5469442

[ref19] Holmqvist S. Chutna O. Bousset L. Aldrin-Kirk P. Li W. Bjorklund T. . (2014). Direct evidence of Parkinson pathology spread from the gastrointestinal tract to the brain in rats. Acta Neuropathol. 128, 805–820. doi: 10.1007/s00401-014-1343-6, 25296989

[ref20] Keshavarzian A. Green S. J. Engen P. A. Voigt R. M. Naqib A. Forsyth C. B. . (2015). Colonic bacterial composition in Parkinson's disease. Mov. Disord. 30, 1351–1360. doi: 10.1002/mds.26307, 26179554

[ref21] Khan S. U. D. Chauhan V. Chaudhary A. A. (2026). The gut-brain-immune axis: multi-omics insights into neurodegenerative and metabolic diseases. Cells 15:1089. doi: 10.3390/cells15121089, 42346116 PMC13297137

[ref22] Kim S. Kwon S. H. Kam T. I. Panicker N. Karuppagounder S. S. Lee S. . (2019). Transneuronal propagation of pathologic alpha-synuclein from the gut to the brain models Parkinson's disease. Neuron 103, 627–641.e7. doi: 10.1016/j.neuron.2019.05.035 PMC6706297 31255487

[ref23] Liu T. W. Chen C. M. Chang K. H. (2022). Biomarker of neuroinflammation in Parkinson's disease. Int. J. Mol. Sci. 23:4148. doi: 10.3390/ijms23084148, 35456966 PMC9028544

[ref24] Ma L. Wang H. B. Hashimoto K. (2025). The vagus nerve: an old but new player in brain-body communication. Brain Behav. Immun. 124, 28–39. doi: 10.1016/j.bbi.2024.11.023, 39566667

[ref25] Mu L. Wang Y. (2025). The role of gut microbiota-derived metabolites in neuroinflammation. Neuroprotection 3, 131–144. doi: 10.1002/nep3.70001, 41383948 PMC12486911

[ref26] Netea M. G. Dominguez-Andres J. Barreiro L. B. Chavakis T. Divangahi M. Fuchs E. . (2020). Defining trained immunity and its role in health and disease. Nat. Rev. Immunol. 20, 375–388. doi: 10.1038/s41577-020-0285-6, 32132681 PMC7186935

[ref27] Nguyen T. T. Baumann P. Tuscher O. Schick S. Endres K. (2023). The aging enteric nervous system. Int. J. Mol. Sci. 24:9471. doi: 10.3390/ijms24119471, 37298421 PMC10253713

[ref28] Panaitescu P. S. Razniceanu V. Mocrei-Rebrean S. M. Neculicioiu V. S. Dragos H. M. Costache C. . (2024). The effect of gut microbiota-targeted interventions on neuroinflammation and motor function in Parkinson's disease animal models-a systematic review. Curr. Issues Mol. Biol. 46, 3946–3974. doi: 10.3390/cimb46050244, 38785512 PMC11120577

[ref29] Saeed S. Quintin J. Kerstens H. H. D. Rao N. A. Aghajanirefah A. Matarese F. . (2014). Epigenetic programming of monocyte-to-macrophage differentiation and trained innate immunity. Science 345:1251086. doi: 10.1126/science.1251086, 25258085 PMC4242194

[ref30] Sampson T. R. Debelius J. W. Thron T. Janssen S. Shastri G. G. Ilhan Z. E. . (2016). Gut microbiota regulate motor deficits and neuroinflammation in a model of Parkinson's disease. Cell 167, 1469–1480.e12. doi: 10.1016/j.cell.2016.11.018, 27912057 PMC5718049

[ref31] Scheperjans F. Aho V. Pereira P. A. Koskinen K. Paulin L. Pekkonen E. . (2015). Gut microbiota are related to Parkinson's disease and clinical phenotype. Mov. Disord. 30, 350–358. doi: 10.1002/mds.26069, 25476529

[ref32] Scheperjans F. Levo R. Bosch B. Laaperi M. Pereira P. A. B. Smolander O. P. . (2024). Fecal microbiota transplantation for treatment of Parkinson disease: a randomized clinical trial. JAMA Neurol. 81, 925–938. doi: 10.1001/jamaneurol.2024.2305, 39073834 PMC11287445

[ref33] Sulzer D. Alcalay R. N. Garretti F. Cote L. Kanter E. Agin-Liebes J. . (2017). T cells from patients with Parkinson's disease recognize alpha-synuclein peptides. Nature 546, 656–661. doi: 10.1038/nature22815 PMC5626019 28636593

[ref34] Tan A. H. Lim S. Y. Chong K. K. A Manap M. A. A. Hor J. W. Lim J. L. . (2021). Probiotics for constipation in Parkinson disease: a randomized placebo-controlled study. Neurology 96, e772–e782. doi: 10.1212/WNL.0000000000010998, 33046607

[ref35] Unger M. M. Spiegel J. Dillmann K. U. Grundmann D. Philippeit H. Burmann J. . (2016). Short chain fatty acids and gut microbiota differ between patients with Parkinson's disease and age-matched controls. Parkinsonism Relat. Disord. 32, 66–72. doi: 10.1016/j.parkreldis.2016.08.019, 27591074

[ref36] Valdetaro L. Ricciardi M. C. Almeida P. P. Stockler-Pinto M. B. Tavares-Gomes A. L. (2026). The enteric nervous system as a mediator of microbiota-gut-brain interactions in Parkinson's disease. J. Neurochem. 170:e70339. doi: 10.1111/jnc.70339, 41486750 PMC12766150

[ref37] van Vuuren M. J. Nell T. A. Carr J. A. Kell D. B. Pretorius E. (2021). Iron dysregulation and inflammagens related to oral and gut health are central to the development of Parkinson's disease. Biomolecules 11:30. doi: 10.3390/biom11010030, 33383805 PMC7823713

[ref38] Wang Q. Luo Y. Chaudhuri K. R. Reynolds R. Tan E. K. Pettersson S. (2021). The role of gut dysbiosis in Parkinson's disease: mechanistic insights and therapeutic options. Brain 144, 2571–2593. doi: 10.1093/brain/awab156, 33856024

[ref39] Wang B. Ma Y. Li S. Yao H. Gu M. Liu Y. . (2023). GSDMD in peripheral myeloid cells regulates microglial immune training and neuroinflammation in Parkinson's disease. Acta Pharm. Sin. B 13, 2663–2679. doi: 10.1016/j.apsb.2023.04.008, 37425058 PMC10326292

[ref40] Wendeln A. C. Degenhardt K. Kaurani L. Gertig M. Ulas T. Jain G. . (2018). Innate immune memory in the brain shapes neurological disease hallmarks. Nature 556, 332–338. doi: 10.1038/s41586-018-0023-4, 29643512 PMC6038912

[ref41] Xu X. Cheng Y. Liu X. Ding W. Zhu Z. Wu L. . (2026). Microbial SCFAs as epigenetic mediators: fine-tuning the gut-brain axis in neurodegenerative disorders. Curr Res Microb Sci. 10:100574. doi: 10.1016/j.crmicr.2026.100574, 41798063 PMC12966653

[ref42] Yuan C. He Y. Xie K. Feng L. Gao S. Cai L. (2023). Review of microbiota gut brain axis and innate immunity in inflammatory and infective diseases. Front. Cell. Infect. Microbiol. 13:1282431. doi: 10.3389/fcimb.2023.1282431, 37868345 PMC10585369

[ref43] Zhao Z. Dong M. Mu Z. Zheng Z. Zhang Z. (2026). Gut-brain axis dysregulation in Parkinson's disease: mechanisms linking microbiota to neuroinflammation and alpha-synuclein pathology. J. Pharm. Anal. 16:101521. doi: 10.1016/j.jpha.2025.101521 PMC13470196 42598050

[ref44] Zhao S. Fu D. Lin Y. Sun X. Wang X. Wu X. . (2025). The role of the microbiome on immune homeostasis of the host nervous system. Front. Immunol. 16:1609960. doi: 10.3389/fimmu.2025.1609960, 40821767 PMC12351403

